# Personalized physical activity recommendations for people with axial spondyloarthritis using wearable activity tracker data: an exploratory study

**DOI:** 10.1007/s00296-024-05755-6

**Published:** 2025-02-26

**Authors:** A.-W de Leeuw, M.A.T. van Wissen, T.P.M. Vliet Vlieland, A.M. van Tubergen, M.G.J. Gademan, M.A.M. Berger, S.F.E. van Weely

**Affiliations:** 1 Department of Orthopaedics, Rehabilitation and Physical Therapy, Leiden University Medical Center, Leiden, the Netherlands; 2 Centre of Expertise Health Innovation, The Hague University of Applied Sciences, The Hague, the Netherlands; 3 Department of Rheumatology, Maastricht University Medical Center, Maastricht, The Netherlands; 4 Care and Public Health Research Institute (CAPHRI), Maastricht University, Maastricht, The Netherlands; 5 Department of Clinical epidemiology, Leiden University Medical Center, Leiden, The Netherlands; 6 Institute of Allied Health Professions, HU University of Applied Sciences, Utrecht, Netherlands

**Keywords:** Axial spondyloarthritis, Physical activity, Sleep, Machine learning

## Abstract

**Objective:**

Benefits of physical activity (PA) on sleep in people with axial SpondyloArthritis (axSpA) are largely unknown. Our aim is to explore the relationships between PA and sleep on both a group level and an individual level using Wearable Activity Trackers (WATs) and machine learning.

**Methods:**

A sample of 64 axSpA participants received a WAT to monitor their PA and sleep. Participants with more than 30 days data of PA and sleep duration were included in the analyses. Spearman’s correlation and the machine learning technique Subgroup Discovery were used to determine relationships between PA during the three prior days and light and deep sleep duration.

**Results:**

Number of daily steps (*n* = 64) was (median (first quartile (Q1) - third quartile (Q3) )) 4026 (1915 - 6549), total sleep (daily light and deep sleep) duration of the participants was 7 h 29 min (6 h 41 min - 8 h 8 min). Nearly 30% (*n* = 18) of the participants were eligible for inclusion in analyses (> 30 days of data). No significant relationships between prior PA and sleep were obtained on a group level. On an individual level, for 8 of the 18 included participants, significant relationships (*p* < 0.05) could be identified between PA during the three prior days and daily sleep duration. These significant relationships differed from participant to participant with a varying qualification of PA (number of steps, intensity level PA) and relevant time window (previous one, two or three days).

**Conclusion:**

Significant relationships between PA and daily sleep duration could be obtained on an individual level with details of the significant relationships varying between participants.

**Registration number:**

Netherlands Trial Register NL8238, included in the International Clinical Trial Registry Platform (ICTRP) (https://trialsearch.who.int/Trial2.aspx?TrialID=NL8238).

**Supplementary Information:**

The online version contains supplementary material available at 10.1007/s00296-024-05755-6.

## Introduction

Axial spondyloarthritis (axSpA) is a chronic rheumatic disease with a progressive course, defined by inflammation of the spine and sacroiliac joints [1]. Joint pain, stiffness and fatigue are major and common symptoms [1]. People with axSpA report that, in addition to pain and stiffness, reduced and disturbed sleep is one of the most important aspects impacting their quality of life [2, 3]. Therefore, sleep is being increasingly recognized as a relevant domain for treatment, and since 2021 sleep has been included as domain for clinical trials in the ASAS-OMERACT core domain set for axSpA [4].

Treatment of axSpA consists of pharmacological and non-pharmacological treatment, whereby the pharmacological treatment typically consists of non-steroidal anti-inflammatory drugs, or biological or targeted synthetic disease modifying anti rheumatic drugs. Non-pharmacological treatment includes education and the promotion of physical activity (PA). PA is any bodily movement produced by skeletal muscles that results in energy expenditure above resting (basal) levels and includes exercise (therapy) [5,6]. Exercise (including supervised therapy), is a form of PA that is planned, structured, and repetitive and has a final or intermediate objective to improve or maintain one or more dimensions of physical functioning and has been proven to be effective in improving physical function, disease activity, mental health and fatigue [7–11].

Many studies have addressed the positive effects of daily PA on sleep in healthy middle-aged and older adults with sleep disturbance [12–14]. Moreover, any type of PA seems to be effective in improving sleep in a diverse range of populations, including people with cancer, mental illness or suffering from other health complaints [15]. However, there is a knowledge gap about the benefits of PA on sleep in people with axSpA. A randomized controlled trial on the effect of high intensity exercise therapy in an axSpA population found no significant improvement in sleep [8]. Furthermore, a systematic review by Runge et al. (2023) on the effect of exercise therapy on sleep in people with arthritis demonstrated the benefits of PA on fatigue in the short term [16]. Still, the review described that evidence for the long-term effects of exercise and any sleep parameter was low and uncertain [16].

In studies assessing the association of PA and sleep, the measurement of PA and the measurement of sleep in people with axSpA have typically been measured with self-reported outcome measures such as the long or short form of the International Physical Activity Questionnaire (IPAQ-LF/SF) and Short Questionnaire to Assess Health (SQUASH) for PA [17–20] and the Pittsburg Sleep Quality Index (PSQI) [3, 8, 16–22] for sleep. Recent technological developments enable assessing PA and sleep simultaneously in real time using wearable activity trackers (WAT) [23]. Until now research has focused on the effectiveness of WATs to improve PA, but also the feasibility (adherence and limitations) of using WATs to promote PA is investigated in trials consisting of people with axSpA or other patient groups [24–27]. However, another important feature of WATs is the collection of substantial longitudinal information simultaneously. This availability of substantial data of individuals opens opportunities to perform analyses not only a group level, but on an individual level as well. In particular, the application of machine learning techniques may facilitate new opportunities to gain valuable insights into the personalization of the associations between PA and sleep in people with axSpA [28] and might offer new directions for personalizing treatment. 

There is lack of knowledge about the effects of PA on sleep in people with axSpA, and associations to date have mainly been investigated using self-reported measures of PA and sleep. New technological advancements like WATs and machine learning can help filling this gap. Therefore, the aim of this study is to explore person-specific associations between PA and sleep in people with axSpA and severe functional limitations by using machine learning and data obtained from WATs.

## Participants and methods

### Design

A secondary analysis was performed with data from a nation-wide randomized clinical trial (RCT), L-EXSPA study (**L**ongterm **EX**ercise therapy in axial **SP**ondylo**A**rthritis) [29, 30]. The study was approved by the Medical Ethical Review Board Leiden-Den Haag-Delft (METC-LDD, NL70093.058.19) and was registered in the Netherlands Trial Register, in the International Clinical Trials Registry Platform (ICTRP, NL8238). Written informed consent was obtained from all participants.

### Participants

The in- and exclusion criteria are equal to the criteria described in an earlier published study protocol of the L-EXSPA study [29, 30]. Eligible individuals were adults (aged ≥ 18 years) with a clinical diagnosis of axSpA (both radiographic and non-radiographic) as confirmed by their rheumatologist. These individuals experienced self-perceived severe limitations in basic daily activities related to self-care (such as dressing and washing), transfers (including getting in and out of bed, rising from a chair or using the toilet), and/or mobility indoors or outdoors. The limitations were directly or indirectly linked to their axSpA, e.g., being caused by persisting or progressive disease activity despite optimal medical treatment and/or severe ankylosis and/or deformities and/or severe comorbidities (e.g., pulmonary or cardiovascular disease, obesity). Additionally, it was determined that their functional limitations were unlikely to improve or be resolved with a brief exercise therapy intervention. Individuals who received individual treatment of a physical therapist or a multidisciplinary team in the setting of a rehabilitation center or rheumatology clinic or center the last three months could not participate in this study. The participant could participate if he/she stopped for a minimum of three months with physical therapy. Patients in need of admission to a hospital, rehabilitation center or rheumatology clinic or other forms of intensive, multidisciplinary care could not participate in the study. Patients who were unable to give informed consent were also not included in the study. 

For our secondary analysis, participants were only included if they received the WAT and used the WAT during the intervention period and recorded more than 30 days data of both PA and sleep, to make sure there are sufficient data for potentially finding statistical significant results on individual level [31].

### General characteristics

At baseline, general characteristic of the participants were collected: Age; sex; weight and height to calculate the Body Mass Index; status of living; level of education; work status; insurance status; duration of complaints; years since diagnosis; radiographic/non-radiographic axSpA; Bath Ankylosing Spondylitis Disease Activity Index (BASDAI); current use of Disease-Modifying Antirheumatic Drugs (DMARDs; bDMARDS, biological Disease-Modifying Antirheumatic Drugs; tsDMARD, targeted synthetic Disease-Modifying Antirheumatic Drugs; csDMARD conventional synthetic Disease-Modifying Antirheumatic Drugs); current use of Non-Steroidal Anti Inflammatory Drugs (NSAIDs), current use of oral glucocorticosteroids; ever smoked; joint surgery history, number of comorbidities. The detailed description of the measurement of the general characteristics and main outcome measures of the L-EXSPA study were described in the earlier published study protocol of the L-EXSPA study [29, 30].

### Physical activity and sleep data

Participants were provided with the Withings PULSE HR^®^ watch (Withings, Lssy les Moulineaux, France) in week 13 of the intervention period to record their daily PA, sleep and assist in guiding participants to increase their levels of PA. In collaboration with the guiding physiotherapists, a daily step goal could be established. The physical therapist also advised wearing the WAT continuously in the 52-week intervention period, removing it only for charging. Daily updates of PA could be seen on the screen of the Withings PULSE HR^®^ watch. To track the change of PA over time, the Withings PULSE HR^®^ had to be connected to the Withings Health Mate App^®^.

The validity and reliability of comparable WATs of Withings (PULSE^®^ and PULSE O2^®^) have been previously studied [32–34], with good results for step counts and sleep duration. As the Withings Pulse HR^®^ is a newer version of these activity trackers, significantly better or at least similar validity and reliability of the device to measure total PA and sleep time were expected.

In this study, step count and the duration of light, moderate and intense activities in seconds were used as variables for the amount of daily PA. Intensity levels of PA were directly obtained from the Withings PULSE HR^®^ by using the predefined settings with the level of PA being derived from the maximum heart rate based on the age of the participant. The total duration of light sleep or deep sleep during both day and night were also directly obtained from the Withings PULSE HR^®^.

### Statistical analysis

The associations between PA and sleep in people with axSpA and severe functional limitations were explored on a daily level. Hence, the analysed data consisted of cases with daily sleep duration and PA during three prior days. Associations in the data were explored using Spearman’s correlations and the machine learning technique Subgroup Discovery [35], methods that will be discussed in more detail later on in this section. Data were analysed using custom made scripts in Python (released October 2021, version 3.10, Python Software Foundation) and Cortana (Windows 10) [36].

Note that in our approach the sample size is equal to the number of days that were monitored and not the number of participants. Hence, the statistical validation of our results is not related to the number of participants and a traditional power analysis is not appropriate here. Traditionally, a power analysis is performed at the start of a study to determine the sample size that is needed to observe an effect. In this study we performed multiple analyses, where the sample size of each analysis is equal to the number of days where we obtained information about both PA and sleep. Therefore, traditional power calculations cannot indicate whether there is sufficient power to draw statistical inferences. Instead, we deploy an alternative approach to assess the statistical significance of our results as discussed in more detail later in this section.

### Construction of dependent and independent variables

Daily light and deep sleep duration were used as dependent variables. A limited set of independent variables was constructed to limit the impact on the statistical significance of our findings in both the Spearman’s correlation and Subgroup Discovery analyses [37]. The total number of steps and duration of light, moderate and intense physical activities were used as quantities of interest to construct the independent variables. We assumed that sleep duration was associated with PA in the three preceding days. Therefore, for all quantities of interest the mean, minimum and maximum values were determined over one, two or three days. By applying the three aggregate functions (mean, minimum and maximum) to the four quantities of interest (number of steps, duration of light, moderate and intense physical activities) in the three time windows (one, two or three days), 28 unique independent variables were obtained for analysis, as the mean, minimum and maximum values for a time window of one day are identical.

### Group-level analysis and personalization of analysis on the individual level

For both the Spearman’s correlation and Subgroup Discovery analysis, results were determined on a group level and an individual level. For the group level analysis, data of all participants were grouped to assess for associations between PA and sleep that apply to all participants. As PA and sleep were vastly different for the participants, independent and dependent variables of each participant were standardized by subtracting the mean and dividing the values by the standard deviation, before merging the data sets. By taking individual differences into account, we avoid that the results of our group analysis merely repeat interpersonal differences in average PA and sleep. For the analysis on the individual level, all participants were considered separately, and potential differences between participants were investigated.

### Methods of analysis

#### Spearman’s correlation

Spearman’s correlations were determined considering all 28 independent variables. Results were determined on a group level and an individual level. The Holm-Bonferroni method was applied to assess the significance of the results [38]. The significance level was set to 0.05 and *p*-values were determined using an unpaired t-test with the null hypothesis that there was no correlation between the variables considered.

#### Subgroup discovery

The machine learning technique Subgroup Discovery [35] was used to find specific subgroups in the data rather than the overall trends obtained from determining Spearman’s correlations. Subgroup Discovery was also performed on group and individual level. Specifically, with Subgroup Discovery, the goal is to find specific subgroups of data that differ significantly from the entire data collection [35]. This difference is quantified by comparing the distribution of the dependent variable, i.e., the light or deep sleep duration. We were interested in finding subgroups for which daily light or deep sleep duration was significantly different from the light or deep sleep duration in the entire data set. Multiple conditions were considered within all independent variables (i.e. mean step count < 1000 or duration of moderate PA > 60 min) with corresponding subgroups containing the cases meeting this considered condition on the independent variable. For example, a possible subgroup would be a collection of all days for which the number of steps on the previous one day was below 5000 with a significantly different light sleep duration compared to the entire data set.

Only subgroups characterized by one condition on a single independent variable and containing between 10% and 90% of the entire data set were used for the analysis to make sure that the results were of sufficient generalizability. The *Subgroup Size* specifies the instances present in the subgroup as percentage of the entire data considered. The *Explained Variance* was used to determine the quality of our subgroup, i.e., the difference of the dependent variable in the subgroup and the instances of the entire data set not present in the subgroup [39]. In each Subgroup Discovery analysis on a group or an individual level, an ordered list of subgroups with descending values for the explained variance was obtained and only the subgroup with the largest explained variance, i.e., the most-pronounced result, was considered.

The significance of the found subgroups in our Subgroup Discovery was determined by using the *Distribution of False Discoveries *[40]. This method assigns a probability to each subgroup quantifying the chance that the found result is only a consequence of considering too many independent variables, a phenomenon also known as the multiple comparison problem [41]. Here, findings were statistically significant if the probability on being a spurious result is less than 5%.

## Results

### Descriptive characteristics

**Fig. 1:**
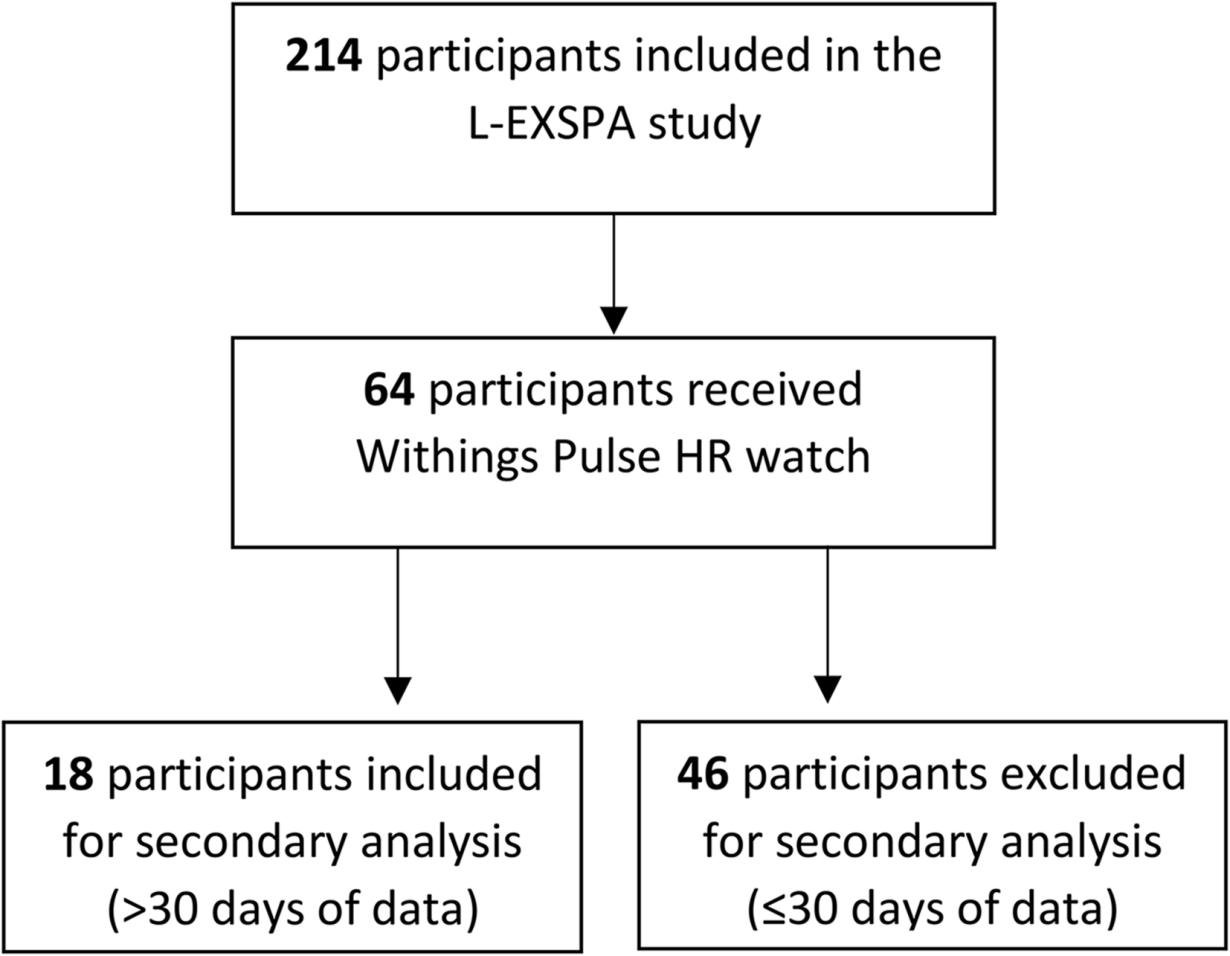
Flowchart of included participants for the secondary analysis

In total 214 people with axSpA and severe functional limitations participated in the L-EXSPA study. A total of 64 participants in the L-EXSPA study received a Withing PULSE HR^®^ watch and collected data, with 18 participants satisfying the requirements for inclusion in the study (> 30 days without missing data for all variables) (Fig. 1). Personal characteristics and global information about the PA and sleep duration of the participants who used a Withing PULSE HR^®^ watch (*n* = 64) and the group considered for the secondary analyses (*n* = 18) can be found in Table 1.

**Table 1:** Characteristics of the population included in this study

Number of participants	Total (*n* = 64)	Not included participants (*n* = 46)	Included participants (*n* = 18)
Female, N (%)	28 (43.8)	20 (43.5)	8 (44.4)
Age in years, median (Q1-Q3)	55.5 (48.0-64.0)	52.0 (46.8-62.5)	60.5 (48.0-66.3)
BMI, median (Q1-Q3)	27.8 (24.7-31.5)	27.9 (24.9-31.2)	27.7 (23.8-33.6)
Self-reported symptom duration (years), median (Q1-Q3)	25.0 (13.3-38.8)	25.0 (11.5-35.0)	22.5 (14.0-41.3)
Time since diagnosis (years), median (Q1-Q3)	12.5 (5.0-25.3) (*N* = 50)	11.0 (5.0-23.8) (*N* = 36)	17.0 (9.0-33.0) (*N* = 14)
Radiographic spondyloarthritis, N (%)	40 (81.6) (*N* = 49)	27 (77.0) (*N* = 35)	13 (92.9) (*N* = 14)
BASDAI score, median (Q1-Q3)	5.0 (4.0-6.3) (*N* = 36)	5.0 (4.0-6.5) (*N* = 25)	5.0 (5.0-6.3) (*N* = 11)
BASFI score, median (Q1-Q3)	6.2 (4.4-8.0) (*N* = 63)	6.1 (4.2-8.2)	6.6 (4.8-7.0)
Number of comorbidities, N (%)	(*N* = 63)	(*N* = 45)	(*N* = 18)
*0 comorbidities*	5 (7.9)	4 (8.9)	1 (5.6)
*1–2 comorbidities*	9 (14.3)	5 (11.1)	4 (22.2)
*3–4 comorbidities*	19 (30.2)	14 (31.1)	5 (27.8)
*>5 comorbidities*	30 (47.6)	22 (48.9)	8 (44.4)
Number of days activity data collected, median (Q1-Q3)	200 (133-200)	200 (97-200)	200 (200-202)
Number of days sleep data collected, median (Q1-Q3)	104 (26-288)	48 (18-166)	268 (214-300)
Median steps per day, median (Q1-Q3)	4026 (1915-6549)	3919 (1507-5733)	5636 (3424-8826)
< 1000, n (%)	7 (10.9)	7 (15.2)	0 (0)
1001–3000, n (%)	14 (21.9)	11 (23.9)	3 (16.7)
3001–6000, n (%)	24 (37.5)	18 (39.1)	6 (33.3)
6001-10.000, n (%)	17 (26.6)	9 (19.6)	8 (44.4)
> 10.000, n (%)	2 (3.1)	1 (2.2)	1 (5.6)
Median duration light intensity activity per day^a^, median (Q1-Q3)	3 h 04 min (2 h 39 min - 4 h 11 min)	3 h 02 min (2 h 42 min - 4 h 04 min)	3 h 27 min (2 h 38 min - 4 h 25 min)
Median duration moderate intensity activity per day^a^, median (Q1-Q3)	0 h 19 min (0 h 11 min - 0 h 39 min)	0 h 17 min (0 h 8 min - 0 h 31 min)	0 h 23 min (0 h 16 min - 0 h 45 min)
Median duration high intensity activity per day^a^, median (Q1-Q3)	0 h 22 min (0 h 13 min - 0 h 40 min)	0 h 15 min (0 h 12 min - 0 h 27 min)	0 h 30 min (0 h 22 min - 0 h 54 min)
Median participants’ total sleep duration per day^a^, median (Q1-Q3)	7 h 29 min (6 h 41 min - 8 h 08 min)	7 h 44 min (6 h 26 min - 8 h 12 min)	7 h 28 min (6 h 47 min - 7 h 46 min)
Median participants’ light sleep duration per day^a^, median (Q1-Q3)	3 h 45 min (2 h 46 min - 4 h 29 min)	3 h 36 min (2 h 34 min - 4 h 29 min)	3 h 49 min (3 h 04 min - 4 h 16 min)
Median participants’ deep sleep duration per day^a^, median (Q1-Q3)	3 h 43 min (3 h 12 min - 4 h 05 min)	3 h 49 min (3 h 08 min - 4 h 09 min)	3 h 38 min (3 h 16 min - 4 h 00 min)

Abbreviations: BMI, Body Mass Index; BASDAI score, Bath Ankylosing Spondylitis Disease Activity Index; BASFI score, Bath Ankylosing Spondylitis Functional Index; Q1, first quartile; Q3, third quartile

^a^ Outliers and participants without data were excluded

The median age in the group considered in the analysis of this study (*n* = 18) was 60.5 (Q1-Q3: 48.0 - 66.3) years with 44.4% being female. 44% of the participants had five or more comorbidities and median disease activity measured with the BASDAI score was 5.0 (Q1-Q3: 5.0 - 6.3) (*n* = 11). Physical functioning measured with the BASFI was 6.6 (Q1-Q3: 4.8 - 7.0). The median total sleeping time in the group was 7 h 28 min (Q1-Q3: 6 h 47 min - 7 h 46 min).

Median wearing time of the Withing PULSE HR^®^ for the 18 participants considered in the analyses of this study was 200 (Q1-Q3: 200 - 202) days for PA and 268 (Q1-Q3: 214 - 300) days for sleep, respectively. The number of days for which both the light and deep sleep duration and all independent variables were registered, ranged from 32 to 173 days. In the analyses, the median number of days available for the 18 participants was 59 (Q1-Q3: 39 - 93). Results of the wearing time of the Withing PULSE HR^®^ watch of the total group of participants are also shown in Table 1.

### Spearman’s correlation

#### Group level analysis

After combining the data of all 18 participants, 1306 days with known light and deep sleep duration and no missing values in the independent variables were available for analysis. Spearman’s correlations between the independent variables related to PA (step count and low, moderate and high intensity PA) and light and deep sleep duration were all small and not statistically significant. The largest correlation was found between duration of light intensity activities on the previous day and light sleep duration (|r| = 0.07).

#### Individual level analysis

Significant associations between the independent variables related to PA (step count and low, moderate and high intensity PA) during the prior three days and light or deep sleep duration were found in five (28%) participants. In Table 2, the significant correlations for the Spearman’s correlation are displayed for these five participants. Results were different for each participant. Independent variables with significant correlations varied in variables reflecting an aspect of PA (number of steps, duration of light, moderate or intense physical activities) and the relevant time window (previous day, prior two or three days).

**Table 2:** Results of the Spearman correlations analysis on the individual level. Overview of significant correlations between daily light or deep sleep duration and independent variables based on physical activity in the preceding days. For each participant and dependent variable (light or deep sleep duration), only correlations with the largest value for the Spearman’s correlation are shown

Participant	Number of observations	Dependent Variable	Independent Variable	Spearman’s correlation	Correlation strength
P01	66	Light sleep duration	Minimum duration of light physical activities in the previous two days	-0.39	Moderate
P02	64	Light sleep duration	Duration of light physical activities in the previous day	-0.48	Moderate
P03	90	Light sleep duration	Maximum duration of intense physical activities previous three days	-0.40	Moderate
P03	90	Deep sleep duration	Duration of moderate physical activities previous one day	0.41	Moderate
P04	97	Light sleep duration	Minimum number of steps previous 3 days	-0.38	Moderate
P05	33	Deep sleep duration	Minimum number of steps previous 3 days	-0.62	Strong

Although specific details varied from participant to participant, we found that PA in the preceding days was negatively correlated with the daily light sleep duration for four participants with significant Spearman’s correlations. The negative correlations imply that more PA in the preceding days was associated with shorter light sleep duration. Significant (negative) associations were seen between minimum duration of light physical activities in the prior two days (*r* = -0.39), duration of light physical activities in the previous day (*r* = -0.48), maximum duration of intense physical activities in the previous three days (*r* = -0.40) or minimum number of steps in the previous three days (*r* = -0.38) and light sleep duration.

For deep sleep duration, results were mixed for two participants. Significant Spearman’s correlations were obtained between duration of moderate physical activities on the prior day and deep sleep duration (*r* = 0.41) for one participant and another participant had a significant association between the minimum number of steps in the previous three days and deep sleep duration (*r* = -0.62).

### Subgroup discovery

#### Group level analysis

For all 18 participants combined, the most-pronounced result was obtained for light sleep duration. Limited PA during the two previous days, i.e., if the number of steps during one of the two previous days was much lower (at least about 3000 steps less than usual), was correlated with different light sleep duration. However, as the *p*-value for this subgroup was 0.13, none of the results obtained from our Subgroup Discovery was significant.

#### Individual level analysis

For 10 (56%) participants, no significant subgroups could be detected for both light and deep sleep duration. Significant subgroups for light and deep sleep duration were obtained for five and four participants, respectively, with one participant having a significant subgroup for both light and deep sleep duration. The descriptions of significant subgroups for light and deep sleep duration are displayed in Tables 3 and 4, respectively.

**Table 3:** Results of the Subgroup Discovery analysis on the individual level. Descriptions of significant subgroups for light sleep duration

Participant	Condition	Subgroup size^a^	Average light sleep duration^a^
P01	Average number of steps in prior 3 days ≤ 1020	11%	1.33
P02	Duration of light physical activities previous day ≤ 72 min	33%	1.14
P04	Minimum number of steps in prior 3 days ≥ 6163	30%	0.91
P06	Average duration of intense physical activities in prior 3 days ≤ 45 min	32%	1.14
P07	Number of steps previous day ≤ 4580	11%	1.70

^a^ Subgroup size and average light sleep duration were determined with respect to the information in the entire dataset of a participant. Hence, average light sleep duration above (below) 1 indicate subgroup with on average longer (shorter) light sleep duration

**Table 4:** Results of the Subgroup Discovery analysis on the individual level. Overview of significant subgroups for deep sleep duration

Participant	Condition	Subgroup size^a^	Average deep sleep duration^a^
P01	Minimum number of steps in prior 3 days ≤ 779	11%	0.74
P03	Duration of moderate physical activities previous day ≥ 62 min	36%	1.11
P05	Minimum number of steps in prior 3 days ≤ 3351	36%	1.30
P08	Duration of light physical activities previous day ≥ 5 h and 46 min	34%	0.86

^a^ Subgroup size and average deep sleep duration were determined with respect to the information in the entire dataset of a participant. Hence, average deep sleep duration above (below) 1 indicate subgroup with on average longer (shorter) deep sleep duration

Description of subsets varied from participant to participant, days with longer light sleep duration were characterized by a limit on the maximal PA in the preceding days for all participants and results for deep sleep duration were mixed. For two participants, a subgroup characterized by a lower limit on the PA in prior days corresponded to longer deep sleep duration. Conversely, an upper limit on the PA in the preceding days were related to longer deep sleep duration for the two other participants.

## Discussion

This study showed that the potential of using machine learning on wearable activity data to assess relevant associations between PA and sleep in people with axSpA and severe functional limitations. After combining data of all participants, no significant relationships between PA and sleep were found on a group level. However, on the individual level significant correlations between prior PA and sleep duration and subgroups of days with significantly different light or deep sleep duration were obtained for specific participants. At the individual level, there was between-person variation in the independent variables (number of steps and duration of light, moderate and intense physical activities) with a significant association between the light or deep sleep duration. This also applied to the description of significant subgroups with abnormal sleep duration.

The total sleep duration in our population was comparable to other studies reporting on sleep duration in people with rheumatic conditions. The obtained total median sleep duration of 7 h is comparable with the mean reported sleep duration of 6–8 h in a rheumatoid arthritis population [42] and slightly longer than the 5–6 h in an axSpA and psoriasis arthritis population [43]. The median daily number of steps per day in our population was 5636 (Q1-Q3: 3424 - 8826). A direct comparison with other studies is difficult, as this study included participants who exhibited severe functional limitations, which may have influenced the median daily step count. However, the daily number of steps of our study was lower compared to previous studies on PA in people with axSpA with a mean number steps per day of 7200 [44], 7197 [45] and 6838 [46]. 

Overall, the outcomes of the Subgroup Discovery on individual level were in line with the Spearman’s correlation analysis on individual level. However, there were also two differences. First, there were three participants with significant subgroups for whom no significant correlations could be detected in the Spearman’s correlation analysis. Second, with our Subgroup Discovery analysis more detailed information about the relationships between independent variables based on prior PA and light or deep sleep duration could be extracted. For example, for participant P04 a negative correlation was obtained between the minimum number of steps in the previous 3 days and light sleep duration. With our Subgroup Discovery approach, this was further specified as shorter light sleep duration was especially encountered if the minimum daily number of steps in the prior 3 days was larger than or equal to 6163.

Up to the best of our knowledge, this is the first study to analyse the associations between PA and sleep in people with axSpA, using data obtained by WATs. A major advantage of this approach is the possibility to collect data on PA and sleep simultaneously. Another strength of our study is that in our analysis using machine learning, we could perform analysis on an individual level. Hereby, we had the possibility to find significant participant-specific results, that we would have missed using conventional techniques analysing the data of all participants together at a group level. This was of crucial importance as significant personalised results could be detected for 8 of the 18 participants included in the analysis. An advantage for the future is the usefulness of the results obtained with this type of analyses. Further research could increase the potential of applying this technology on sleep and PA data to facilitate health care professionals providing person-specific advice on improving PA and sleep.

 There are limitations of our study that need to be considered. Most importantly, for several reasons the generalizability of the findings in our study is limited. First, the results presented here are only applicable to the axSpA people with severe functional limitations considered in this study and the question remains how our results translate to the whole group of people with axSpA or other rheumatic diseases. Second, the sample size was relatively small as the WATs were only used in the intervention group of the RCT and therefore many participants had insufficient data for inclusion in the analyses. Third, the data collected with the Withing PULSE HR®watch were no outcome measures in the primary study (i.e. the L-EXSPA study). 

A second limitation of this study was the quality of the data. Comparable results for daily number of steps and sleep duration to previous studies with similar populations suggests the data are reliable. However, the accuracy of WATs and in particular the reliability and validity of sleep staging on superficial level (e.g., Light vs. Deep) remain a point of attention [32–34]. To the best of our knowledge, there is no information available yet on the reliability of the outcome, light, moderate and intense activities measured by the Withings activity tracker. Nonetheless, the systematic review of Gorzelitz et al. (2020) suggests that (commercially) available wearable activity trackers are valid for measuring moderate-to vigorous-intensity physical activity [47].

A third limitation of this study was the large variation in wearing time of the WAT. As a result, the participants with a longer wearing time collected more data. Especially in the group level analysis, this might have influenced the results as some patients contributed more than others. Despite the limitations, this exploratory study provides direction for future research on the relationships between PA and daily sleep. More rigorous data collection, emphasis on participants commitment in using the WAT and adding data collection and analyses with different indices such as self-reported measures for the evaluation of sleep quality may be of added value.

In conclusion, this exploratory study showed the benefits of data acquisition via WATs and using machine learning techniques to assess the associations between PA and sleep in people with axSpA and severe limitations in functioning. This approach revealed significant relationships between PA and sleep for 8 of the 18 participants that would have been missed by grouping all participants together. In near future, this study should be repeated in a larger and broader group of participants with axSpA (or rheumatic conditions) to assess the generalizability of our findings and before this technique can be used for person-specific guidance.

## Electronic Supplementary Material

Below is the link to the electronic supplementary material.

Supplementary Material 1

## Data Availability

The data underlying this article will be shared on reasonable request to the corresponding author.
